# MEK1/2 inhibition enhances the radiosensitivity of cancer cells by downregulating survival and growth signals mediated by EGFR ligands

**DOI:** 10.3892/ijo.2013.1890

**Published:** 2013-04-10

**Authors:** EUN JOO CHUNG, MARY ELLEN URICK, NAAMIT KURSHAN, WILLIAM SHIELD, HIROAKI ASANO, PAUL D. SMITH, BRADLEY S. SCROGGINS, JEFFREY BURKEEN, DEBORAH E. CITRIN

**Affiliations:** 1 Section of Translational Radiation Oncology, Radiation Oncology Branch, National Institutes of Health, Bethesda, MD, USA;; 2 Department of Cancer and Thoracic Surgery, Okayama University Graduate School of Medicine and Dentistry, Okayama, Japan;; 3 Cancer and Infection Research Area, AstraZeneca, Alderley Park, Macclesfield, UK

**Keywords:** radiation, transforming growth factor-α, AZD6244, selumetinib, Ras

## Abstract

The inhibition of the Ras/mitogen-activated protein kinase (Ras/MAPK) pathway through the suppression of mutated Ras or MAPK/extracellular signal-regulated kinase 1/2 (MEK1/2) has been shown to sensitize tumor cells to ionizing radiation (IR). The molecular mechanisms of this sensitization however, are not yet fully understood. In this study, we investigated the role of transforming growth factor-α (TGF-α) in the radiosensitizing effects of selumetinib, a selective inhibitor of MEK1/2. The expression of epidermal growth factor receptor (EGFR) ligands was assessed by ELISA in both Ras wild-type and Ras mutant cells that were exposed to radiation with or without selumetinib. The effects of selumetinib on the TGF-α/EGFR signaling cascade in response to radiation were examined by western blot analysis, clonogenic assay and by determing the yield of mitotic catastrophe. The treatment of cells with selumetinib reduced the basal and IR-induced secretion of TGF-α in both Ras wild-type and Ras mutant cell lines *in vitro* and *in vivo*. The reduction of TGF-α secretion was accompanied with a reduction in phosphorylated tumor necrosis factor-α converting enzyme (TACE) in the cells treated with selumetinib with or without IR. The treatment of cells with selumetinib with or without IR inhibited the phosphorylation of EGFR and check-point kinase 2 (Chk2), and reduced the expression of survivin. Supplementation with exogenous TGF-α partially rescued the selumetinib-treated cells from IR-induced cell death, restored EGFR and Chk2 phosphorylation and increased survivin expression. These data suggest that the inhibition of MEK1/2 with selumetinib may provide a mechanism to sensitize tumor cells to IR in a fashion that prevents the activation of the TGF-α autocrine loop following IR.

## Introduction

The uncontrolled growth of cancer cells is frequently driven by the acquisition of activating mutations in growth-promoting genes (1,2), inactivating mutations in growth-inhibitory genes and epigenetic abnormalities that lead to the altered expression of growth-modulating proteins (1,3). These genetic or/and epigenetic changes can result in the constitutive activation of signaling pathways that lead to uncontrolled proliferation, metastasis and resistance to anticancer therapy.

The epidermal growth factor receptor (EGFR)/Ras pathway is a key signaling cascade involved in cell proliferation, stress response, migration and cell cycle progression (4,5). The constitutive activation of the EGFR/Ras pathway may result from activating mutations in *Ras (KRAS, HRAS, NRAS)* and *RAF* and via the mutation or overexpression of EGFR (6–9). Cells harboring a constitutively activated EGFR/Ras pathway via mutant Ras or Raf are known to be more sensitive to MAPK/extracellular signal-regulated kinase (ERK) (MEK) inhibitors (10–12); however, sensitivity in Ras and Raf mutant cells is not uniform and even Ras and Raf mutant cells may exhibit resistance to MEK inhibition (10,12,13). Collectively, these data suggest that sensitivity to these agents may be complex and dependent on a host of mutations, gene expression patterns, or the inherent dependence of the cell to aberrant signaling through the Ras/mitogen-activated protein kinase (Ras/MAPK) pathway (14).

We previously reported that the treatment of 3 human cancer lines with the MEK1/2 inhibitor, selumetinib (AZD6244, ARRY-142886), sensitized the cells to ionizing radiation (IR) (15). We observed an increase in mitotic catastrophe in the cell lines exposed to selumetinib prior to IR compared to those that were treated with IR alone. To further evaluate the mechanisms by which the inhibition of the Ras/MAPK pathway results in radiosensitization, we focused on autocrine growth factors that signal through EGFR following radiation. Ligands of EGFR have been shown to be secreted by different types of cancer cells following IR, resulting in the autocrine activation of the EGFR/Ras/MEK/MAPK pathways which can protect irradiated cells from IR-induced death (16–18).

In the present study, we investigated the role of secreted transforming growth factor-α (TGF-α), an EGFR ligand, on the radiosensitization mediated by selumetinib in A549 cells (KRAS mutant), in DU145 transfectants expressing wild-type Ras, and in DU145 transfectants harboring a KRAS mutation. We hypothesized that the interruption of MAPK signaling with selumetinib in KRAS-transformed tumor cells would decrease the production of TGF-α and prevent the secondary activation of the EGFR downstream signaling pathways, a known resistance mechanism following the inhibition of mutant Ras (19). Our data support the notion that inhibiting MEK provides a means to sensitize cells to IR through the interference of Ras/MAPK and TGF-α signaling via EGFR.

## Materials and methods

### Cell lines and treatment

The A549 non-small cell lung cancer (NSCLC) and DU145 (prostate cancer) cell lines were obtained from American Type Culture Collection (ATCC; Manassas, VA). All cell lines were verified by DNA fingerprinting and confirmed to be mycoplasma-free by ATCC. Cells were cultured in RPMI-1640 medium (ATCC), supplemented with 5% fetal bovine serum (Hyclone, Logan, UT). Cells were maintained at 37°C, 5% CO2. Selumetinib, provided by AstraZeneca (Macclesfield, UK), was reconstituted in DMSO and stored at −20°C. Recombinant human TGF-α and anti-TGF-α antibodies were purchased from R&D Systems (Minneapolis, MN) and EMD Chemicals (Gibbstown, NJ), respectively. Cultures were irradiated using a Pantak (Solon, OH) X-ray source at a dose rate of 1.55 Gy/min.

### Plasmid and transfection

DU145 cells were transfected with an empty vector or a plasmid expressing a hemagglutinin (HA)-tagged *KRAS2A G12V* (Biomyx Technology, San Diego, CA) using the Nucleofector transfection system (Amaxa Inc., Gaithersburg, MD) according to the manufacturer’s instructions. Transfectants were placed under selection with G418 (Invitrogen, Carlsbad, CA) and pooled stable cell lines (DU145 vec, DU145 mut) were established. Transgene expression was confirmed by western blot analysis using a HA antibody.

### Clonogenic assays

Cell cultures were trypsinized to generate a single cell suspension and a specified number of cells were seeded into 6-well tissue culture plates. After allowing 6 h for attachment, the cells were incubated with 250 nM selumetinib or DMSO (vehicle control) for 16 h prior to IR. Anti-TGF-α antibody (final concentration, 1 *μ*g/ml) was added 30 min prior to IR to neutralize endogenous TGF-α in the culture medium and recombinant TGF-α (10 pg/ml) was added to the cultures immediately before IR. Twelve to 14 days after seeding, colonies were stained with crystal violet, the number of colonies containing at least 50 cells was determined, and the surviving fractions were calculated. Survival curves were generated after normalizing for cytotoxicity generated by treatment alone for each independent experiment. Data presented are the means ± SEM from at least 3 independent experiments. The dose enhancement factor (DEF) was determined by taking the ratio of the dose leading to a surviving fraction of 0.1 for untreated versus treated cells (for each condition: neutralizing antibody, selumetinib, or selumetinib + TGF-α).

### Mitotic catastrophe

The presence of fragmented nuclei was used as the criteria for defining cells undergoing mitotic catastrophe (15,20). To visualize nuclear fragmentation, cells were fixed with methanol for 15 min at −20°C and stained with anti-α-tubulin monoclonal antibody (T6199; Sigma-Aldrich, St. Louis, MO) followed by staining with FITC-conjugated secondary antibody (Jackson ImmunuoResearch Laboratories Inc., West Grove, PA). Nuclei were counterstained with DAPI. A total of 150 randomly selected cells were analyzed from each treatment group and photographed under an epifluorescence microscope. Nuclear fragmentation was defined as the presence of >2 distinct nuclear lobes within a single cell.

### Western blot analysis

Cell extracts were prepared using RIPA buffer (Pierce, Rockford, IL) containing protease inhibitors (Roche Applied Science, Indianapolis, IN) and phosphatase inhibitors (Sigma-Aldrich), followed by measurement of protein concentrations by the Bradford method (Bio-Rad, Hercules, CA). Equal amounts of protein were subjected to western blot analysis, and were probed with the primary antibody indicated. ImageQuant software (GE Healthcare, Pittsburgh, PA) was used to evaluate the relative expression of each target protein normalized to actin.

### ELISA

Culture supernatants were obtained from the cells (1×10^4^/100 *μ*l) pre-treated with/without selumetinib at various time-points after IR as indicated. In order to obtain lysates from A549 tumors, tissue pieces were collected from mice treated as indicated, and then homogenized in RIPA buffer containing protease inhibitors. Soluble proteins were collected by centrifugation (10,000g × 10 min) and followed by the measurement of protein concentrations by the Bradford method (Bio-Rad). The levels of soluble TGF-α, amphiregulin and heregulin in the culture supernatants or lysates from the tumor tissues were assessed using the human Quantikine ELISA kit for TGF-α and the human DuoSet ELISA kits for amphiregulin and heregulin (R&D Systems) according to the manufacturer’s instructions.

### Mouse xenograft model

Animal experiments were conducted in accordance with the principles and procedures outlined in the National Institutes of Health (NIH) Guide for the Care and Use of Laboratory Animals. Nude mice, 4-6 weeks old [National Cancer Institute (NCI), Frederick, MD], were injected subcutaneously with A549 cells (1×10^6^/100 *μ*l PBS/mouse) on the lateral aspect of the rear leg. When tumors reached 250 mm^3^ mice were treated with selumetinib (50 mg/kg) or the vehicle control by oral gavage. Restrained mice were irradiated to the hind leg 4 h after selumetinib administration using a Pantak irradiator. Tumor tissue was excised at the indicated time-points. For ELISA, tumors were homogenized in RIPA buffer containing protease inhibitors to extract soluble proteins. For immunohistochemistry, tumors were fixed with 10% neutral-buffered formalin and embedded in paraffin.

### Immunohistochemistry

Sections (6-*μ*m-thick) mounted on poly-L-lysine coated glass slides were deparaffinized, rehydrated, incubated in 3% H_2_O_2_ for 5 min, and boiled for 30 min in 10 mM sodium citrate buffer (pH 6.0; Vector Laboratories, Burlingame, CA). TGF-α expression was assayed with an indirect immunoperoxidase method (ImmPRESS, Vector Laboratories) using anti-TGF-α polyclonal antibody (1:50 dilution; Abcam, Cambridge, MA). Following treatment with 3,3-diaminobenzidine (Roche) sections were counterstained in hematoxylin, dehydrated through graded alcohols, cleared in xylenes, and mounted in Permount (Sigma-Aldrich).

### Statistical analysis

*In vitro* experiments were repeated thrice, and statistical analysis was carried out using a Student’s t-test. Data are presented as the means ± SD. A probability level of P<0.05 was considered to indicate a statistically significant difference.

## Results

### Exposure to selumetinib alters the activation of EGFR after radiation

**Figure 1:**
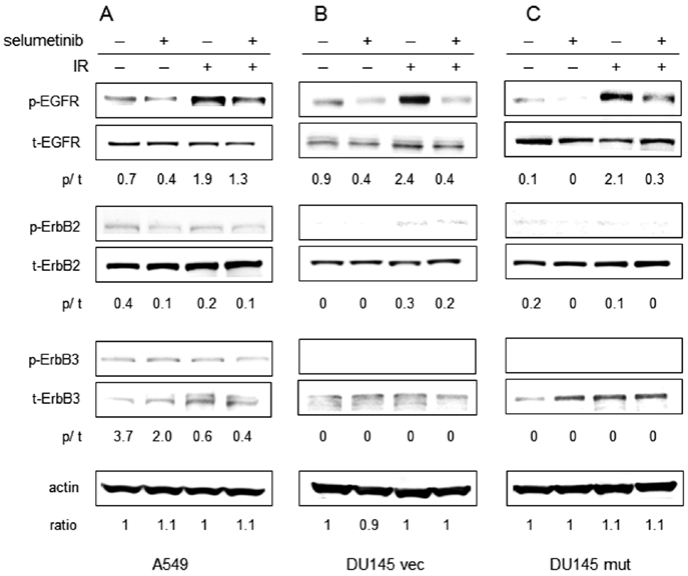
Expression profiles ErbB receptor family in response to IR with/without selumetinib. (A) A549, (B) DU145 vec and (C) DU145 mut cells were exposed to 250 nM selumetinib or the vehicle control for 16 h, irradiated, and harvested 24 h after IR (4 Gy) for immunoblotting. To evaluate the expression levels of phosphorylated or total ErbB receptors, immunoblot assay was performed.

EGFR, ErbB2 and ErbB3 are members of the ErbB receptor family of tyrosine kinases expressed on the cell surface. The heterodimerazation or homodimerization of these receptors plays an important role in the association of EGFRs with ligands and downstream signaling pathways. To investigate whether the exposure to selumetinib alters the magnitude of ErbB receptor activation in response to radiation in our cell lines, the level of phosphorylation of each receptor was examined at 24 h following radiation in the A549, DU145 vec and DU145 mut cells (Fig. 1). As expected, irradiation resulted in the increased phosphorylation of EGFR (Tyr845) in all 3 cell lines. There was no evidence of the altered phosphorylation of ErbB2 (Tyr1221/1222) and ErbB3 (Tyr1197) following irradiation. The phosphorylation of EGFR decreased significantly following treatment with selumetinib in the presence or absence of IR in all 3 cell lines. Treatment with selumetinib moderately reduced the phosphorylation of ErbB2 in the A549 and DU145 mut cells (both Ras mutants) with or without IR. ErbB3 phosphorylation appeared minimally affected by selumetinib treatment in A549 cells and was not detectable in the DU145 vec or DU145 mut cells.

### Selumetinib inhibits EGFR ligand secretion through the downregulation of metalloproteinase tumor necrosis factor (TNF)-α converting enzyme (TACE) activation

**Figure 2:**
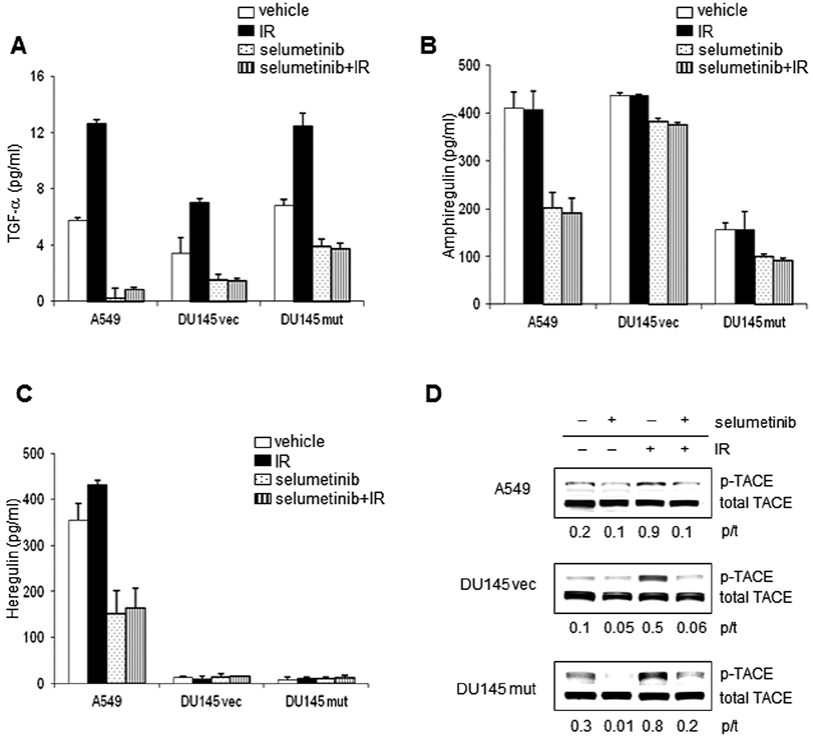
Effects of selumetinib on EGFR ligand secretion in response to IR. (A–C) Levels of soluble EGFR ligands: Supernatants were collected 24 h after IR (4 Gy) from cell cultures pre-treated with the vehicle or 250 nM selumetinib. ELISA was performed to assess the levels of soluble (A) TGF-α, (B) amphiregulin and (C) heregulin. TGF-α and heregulin secretions were increased in response to IR in A549, DU145 vec and DU145 mut cells. Selumetinib inhibited TGF-α, amphiregulin and heregulin secretion with/without IR in each cell line. Columns, average; bars, SD. (D) Effects of selumetinib on TACE activation: Levels of phosphorylated TACE were assessed by immunoblotting. The levels of phosphorylated TACE were increased by IR and decreased with selumetinib treatment in all 3 cell lines.

TGF-α, amphiregulin and heregulin are soluble factors which have been linked to radiation resistance in *Ras*-transformed cells (17,21). To investigate whether the inhibition of MEK can alter the elaboration EGFR ligands, levels of soluble TGF-α, heregulin and amphiregulin were assessed by ELISA in the A549, DU145 vec and DU145 mut cells treated with IR (4 Gy) and/or selumetinib (Fig. 2). TGF-α secretion was induced by IR in all 3 cell lines. DU145 mut cells secreted significantly higher levels of TGF-α than DU145 vec cells, at a level similar to the A549 cell line. MEK inhibition reduced TGF-α secretion in all 3 cell lines, under irradiated and unirradiated conditions (Fig. 2A). In all the cell lines, treatment with selumetinib reduced TGF-α secretion after IR to levels lower than those observed under untreated conditions. Amphiregulin secretion was not induced by radiation in the 3 cell lines tested; however, basal levels of amphiregulin secretion were inhibited by MEK inhibition (Fig. 2B). Although the induction of heregulin secretion in response to IR was statistically significant compared to the control (p<0.008), the relative increase was minimal. Furthermore, the increase in phosphorylation of ErbB3 in irradiated A549 cells compared to the unirradiated controls was minimal. Basal and radiation-induced levels of heregulin were markedly inhibited by selumetinib (Fig. 2C).

The secretion of soluble EGFR ligands is known to be regulated by TACE, also known as ADAM-17 (22,23). The activation (phosphorylation) of TACE occurred after IR in all 3 cell lines (Fig. 2D). Treatment with selumetinib was sufficient to inhibit the phosphorylation of TACE in the presence or absence of IR in all 3 cell lines. The activation of TACE has been reported to require an association with phosphorylated ERK1/2 (24,25). The association between TACE and phosphorylated ERK1/2 was increased by 1.8-fold, 4 h after IR in the A549 cells compared to the unirradiated cells (data not shown). Treatment with selumetinib decreased this association following IR to levels lower than those observed in the controls, possibly due to a reduction in the amount of phosphorylated ERK.

### TGF-α autocrine signal is required for cancer cell survival and xenograft tumor growth following radiation

**Figure 3:**
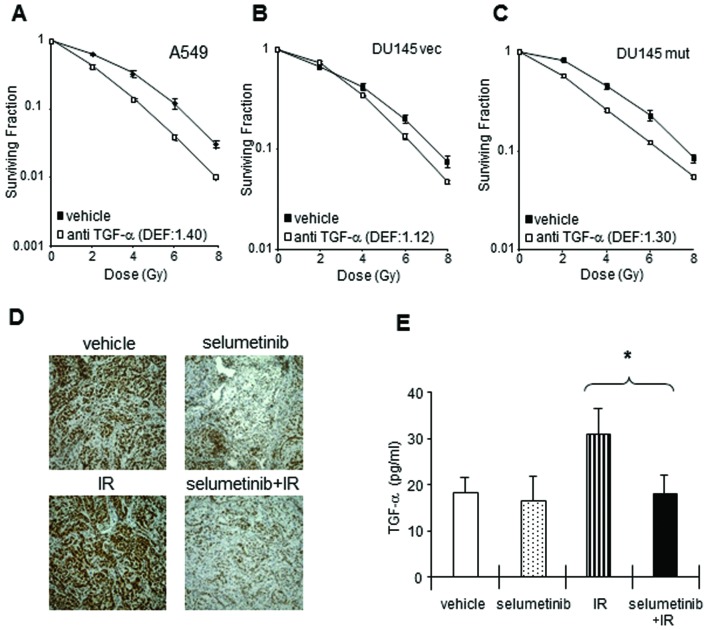
Increased TGF-α expression in response to IR is required for A549 cell survival in *in vitro* cultures and *in vivo* tumors. (A–C) Neutralizing anti-TGF-α antibody decreased the clonogenic survival in tumor cells exposed to IR. (A) A549, (B) DU145 vec and (C) DU145 mut cells were exposed to neutralizing TGF-α antibody (final concentration, 1 *μ*g/ml), followed 30 min later by IR, and incubated at 37°C with 5% CO_2_. Colony-forming efficiency was determined 10 to 14 days later and survival curves were generated after normalizing for cell killing by anti-TGF-α alone. Clonogenic survival after IR was inhibited by the elimination of soluble TGF-α in A549, DU145 vec and DU145 mut cells. The data represent the means of 3 independent experiments. PE, plating efficiency with selumetinib; DEF, dose enhancement factor. Points, mean; bars, + SE. (D–E) Effects of selumetinib on TGF-α induction in response to IR in A549 xenograft tumors. When A549 tumors reached 250 mm^3^ in size, the mice were randomized into 4 groups: vehicle, selumetinib, IR (3 Gy), or selumetinib plus IR. Selumetinib was administered by mouth (oral gavage) in a single dose of 50 mg/kg. IR (3 Gy) was delivered 4 h after selumetinib treatment. Tumors were harvested at 24 h after IR and subjected to TGF-α IHC (D) or ELISA (E). The levels of endogenous TGF-α were increased 24 h after IR in A549 xenografts. Selumetinib treatment decreased the level of endogenous TGF-α with/without IR in A549 tumors. Columns, mean; bars, ± SE.

To investigate the importance of radiation-induced TGF-α for clonogenic survival in our cell lines, a neutralizing antibody against TGF-α was added to the cultures 30 min prior to IR. As shown in Fig. 3, the neutralization of endogenous TGF-α decreased clonogenic survival in the A549, DU145 vec and DU145 mut cells, suggesting that TGF-α increases the survival of irradiated tumor cells. The dependency on TGF-α in the post-irradiation setting was greatest in the *KRAS* mutant cells with TGF-α neutralization providing a DEF of 1.4 for the A549, 1.3 for the DU145 mut, and 1.12 for the DU145 vec cells.

We previously reported an enhancement in radiosensitivity with MEK inhibition *in vivo* in an A549 xenograft model (15). To determine whether MEK inhibition is capable of reducing TGF-α elaboration *in vivo*, the levels of TGF-α following treatment with IR and/or selumetinib were assessed by immunohistochemistry and ELISA in A549 xenografts (Fig. 3E). The total expression of TGF-α was lower in the xenografts from mice treated with selumetinib alone or selumetinib in combination with IR compared to basal levels. Given the heterogeneity of the expression of TGF-α observed after immunohistochemical assay (Fig. 3E), further confirmation of a reduction in TGF-α expression was achieved with the more quantitative approach of ELISA. TGF-α expression in the xenograft tumors was increased 24 h after IR. Pre-treatment with selumetinib 4 h prior to IR resulted in decreased TGF-α expression to a level similar to that achieved with selumetinib alone.

### TGF-α partially rescues tumor cells from selumetinib-mediated radiation sensitization

**Figure 4:**
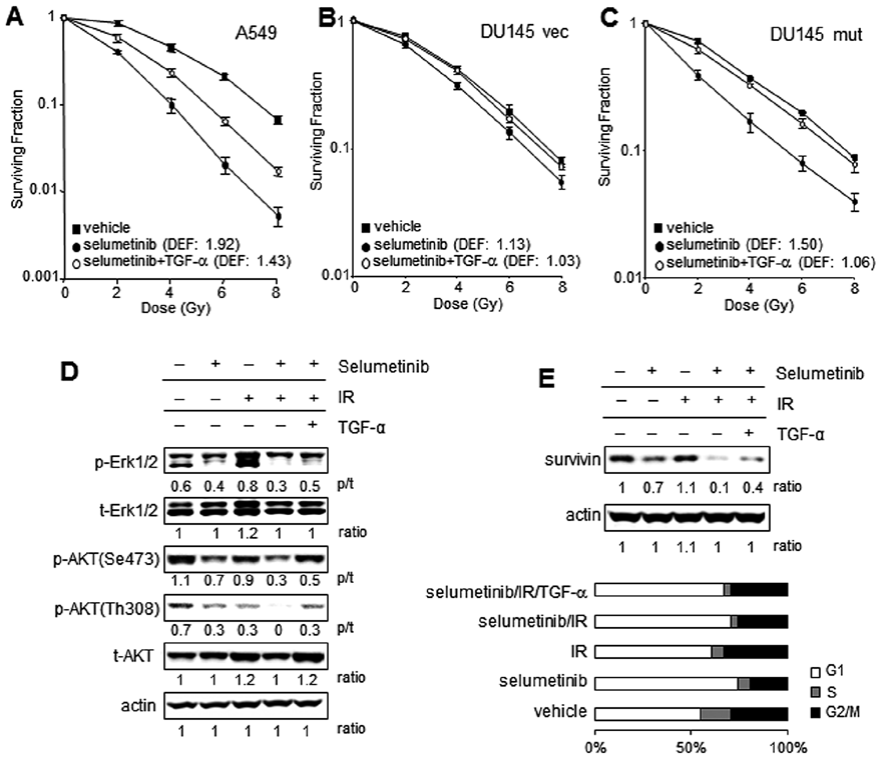
Exogenous TGF-α supplementation restores EGFR downstream signaling after selumetinib-mediated inhibition in irradiated tumor cells. (A–C) Clonogenic assays: Cells were exposed to 250 nM selumetinib or the vehicle control for 16 h, irradiated with graded doses of X-rays and supplemented with recombinant human TGF-α (rhTGF-α) (10 pg/ml) or PBS immediately after IR. Colony-forming efficiency was determined 10 to 14 days later and survival curves were generated after normalizing for cell killing by selumetinib alone. The data represent the means of 3 independent experiments. Significant sensitizations to IR with selumetinib were observed in (A) A549 and (C) DU145 mut cells compared to (B) DU145 vec cells. Exogenous TGF-α partially rescued the A549 cells and the DU145 transfectant cells almost completely from selumetinib-induced radiosensitization. DEF, dose enhancement factor; points, mean ± SE. (D and E) Restoration of EGFR downstream signals by exogenous TGF-α. A549 cells were exposed to 250 nM selumetinib or the vehicle control for 16 h, irradiated and harvested 24 h following IR (4 Gy) for immunoblotting. To evaluate the downstream signaling after EGFR activation by TGF-α binding, the levels of phosphorylated AKT and ERK1/2 were assessed in lysates obtained from the cells treated with various combinations of IR, TGF-α and selumetinib. (D) The phosphorylation of ERK1/2 was increased by IR, while the phosphorylation of AKT was slightly decreased by IR. The effects of the inhibition by selumetinib were assessed in the cells treated with or without IR. The addition of TGF-α to the selumetinib-treated cells partially restored the phosphorylation of AKT and ERK1/2. The levels of survivin, and EGFR/MAPK downstream target molecule were also investigated. (E) Survivin expression was partially decreased by selumetinib, and significantly by the combination treatment with IR. Exogenous TGF-α reversed the inhibitory effects on survivin expression in A549 cells treated with selumetinib and IR. As survivin expression is related to the cell cycle, cell cycle profiles of cells treated with IR, selumetinib and selumetinib/IR were investigated 24 h after IR. The expression levels of survivin were not a result of the number of cells in each phase of the cell cycle between the cells treated with selumetinib alone and selumetinib/IR.

The results presented above suggest that the radiation-induced secretion of TGF-α may act as a survival factor, and that MEK inhibition may block the elaboration of basal and radiation-induced TGF-α levels. To confirm that TGF-α remains an important survival factor following IR in the setting of MEK inhibition, clonogenic assays were performed with selumetinib with or without the addition of TGF-α. Radiosensitization with selumetinib was evident to a greater extent in KRAS mutant cell lines with a DEF of 1.9 in the A549 cell line and 1.5 in DU145 mut (DEF of 1.5) compared to 1.13 in the DU145 vec line. The addition of exogenous TGF-α rescued all the cell lines from selumetinib-enhanced radiation-induced cytotoxicity (Fig. 4A–C) with almost complete rescue in the DU145 vec and DU145 mut lines and partial rescue in the A549 cell line.

To further evaluate the molecular events underlying the ability of TGF-α to rescue cells from radiation sensitization by MEK inhibition, the A549 cell line was investigated. Our primary hypothesis was that TGF-α is depleted by MEK inhibition and recovery to post-irradiation levels activates the EGFR pro-survival signaling pathway which permits the rescue of irradiated cells. To examine whether the addition of exogenous TGF-α restores the EGFR signaling altered by selumetinib in irradiated A549 cells, the phosphorylation of EGFR and the downstream molecules, ERK1/2 and AKT, and the expression levels of survivin were assessed by immunoblotting (Fig. 4D and E). The exposure to radiation increased phosphorylated ERK1/2, but decreased the phosphorylation of AKT at serine 473 and threonine 308 in A549 cells at 24 h. Treatment with selumetinib decreased the levels of ERK1/2 phosphorylation and AKT phosphorylation in the presence or absence of IR. The addition of TGF-α to the cells treated with selumetinib and IR partially restored the phosphorylation of ERK1/2, although it completely recovered AKT phosphorylation inhibited by selumetinib in irradiated A549 cells. This suggests that ERK1/2 was inhibited continuously after the addition of TGF-α due to selumetinib remaining in the culture. Survivin is known to be a prosurvival molecule, a known downstream target of the MAPK/ERK pathway and is involved in the progression of mitosis. As shown in Fig. 4E, survivin expression was markedly inhibited by the combination treatment with selumetinib and IR compared to that observed after single treatment with IR or selumetinib. The addition of TGF-α partially restored the expression of survivin in the A549 cells exposed to selumetinib and IR. The expression of survivin is related to the cell cycle, with reported dominant expression in the G2/M phase (26). Cell cycle analysis confirmed that there was no marked alteration in the percentage of cells in G2/M following treatment with selumetinib and/or TGF-α in irradiated A549 cells, suggesting that the increased survivin expression was not a result of cell cycle changes (Fig. 4E).

### TGF-α supplementation reduces mitotic catastrophe after IR in selumetinib-treated tumor cells

**Figure 5:**
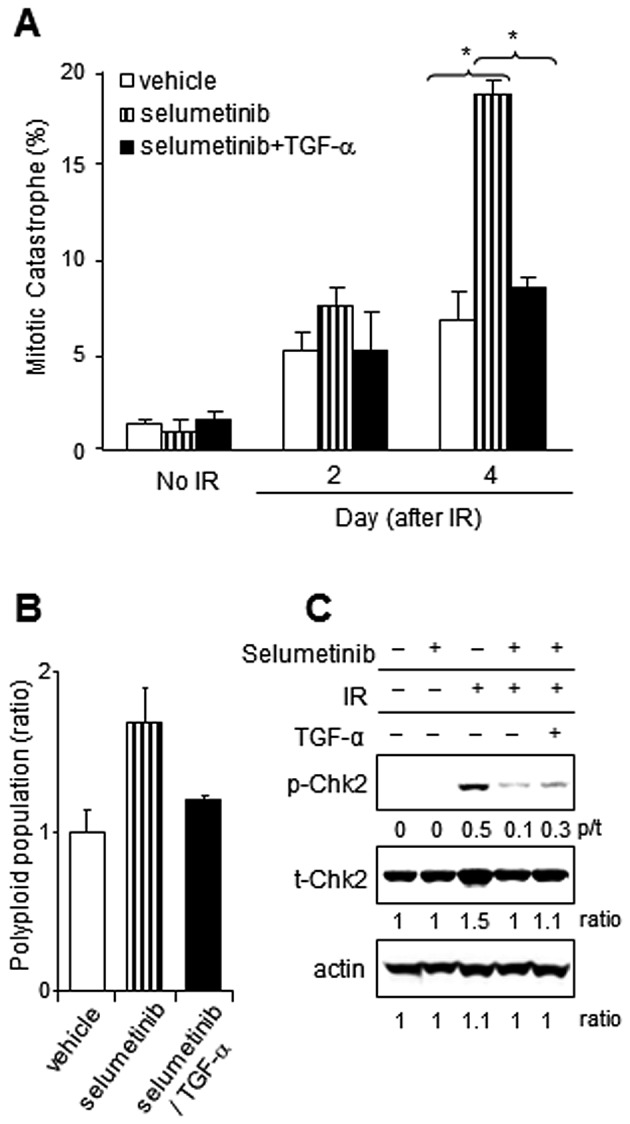
Effects of TGF-α on enhanced mitotic catastrophe induced by selumetinib after radiation in A549. (A) Mitotic catastrophe: Cells growing in chamber slides were exposed to selumetinib (250 nM) or the vehicle control, IR (4 Gy), with or without the addition of TGF-α and fixed at the specified times for immunocytochemical analysis for mitotic catastrophe. Nuclear fragmentation was evaluated in 150 cells per treatment from 5 different fields. Nuclear fragmentation was defined as the presence of ≥2 distinct lobes within a single cell. TGF-α supplementation reduced mitotic catastrophe in A549 cells treated with selumetinib and IR. Columns, mean; bars, SE. Nuclear fragmentation was defined as the presence of ≥2 distinct lobes within a single cell. ^*^P<0.05 according to the Student’s t-test (selumetinib vs. selumetinib + TGF-α). (B) Polyploid population: Polyploid cells containing abnormal DNA (>4 n) were detected by flow cytometry in A549 cells treated as indicated at 24 h after IR exposure. Polyploidy after IR exposure was enhanced by selumetinib, however TGF-α addition reduced the level of polyplod population down to the level of IR alone. (C) Western blot analysis for phosphorylated Chk2: Chk2 is known a regulator of mitotic catastrophe. The level of activated Chk2 was investigated by immunoblotting in A549 cells treated with selumetinib (250 nM), IR (4 Gy) and rhTGF-α (10 pg/ml) 24 h following IR.

In our previous study, an increase in the number of cells undergoing mitotic catastrophe was defined as an important mechanism of cell death after the combined treatment with selumetinib and IR compared to either treatment alone (15). In the present study, the mitotic catastrophe induced by the combination of selumetinib and IR was inhibited significantly by TGF-α supplementation in A549 cells (Fig. 5A). The increase in the polyploid population with selumetinib supplementation was confirmed at 24 h after IR exposure in A549; however, it was reduced by the addition of TGF-α (Fig. 5B). To evaluate the mechanism by which TGF-α protects cancer cells from mitotic catastrophe, we examined the expression and phosphorylation of checkpoint kinase 2 (Chk2), which is known as both a regulator of mitotic catastrophe (27) and as a kinase that phosphorylates survivin in response to DNA damage (34). As observed in Fig. 5C, the phosphorylation of Chk2 was detected in irradiated A549 cells, but not in unirradiated cells. The IR-induced Chk2 phosphorylation was inhibited by selumetinib treatment and was partially restored with the addition of TGF-α.

## Discussion

The acute effects of IR-induced DNA damage have been well documented. Since DNA double-strand breaks (DSBs) are considered to be a lethal event following IR (28,29), much emphasis has been placed on the evaluation of DNA repair and events occurring early after IR, when novel radiation modifiers are evaluated. We previously reported the radiosensitizing effects of selumetinib in human cancer cell lines of 3 different histologies (15). We observed enhanced sensitization to radiation with selumetinib treatment in KRAS mutant cell lines in this, as well as our previous study. We also observed that prolonged post-IR exposure to selumetinib increased the degree of sensitization in all 3 cell lines (data not shown). These findings suggest that constitutively active KRAS and prolonged MEK/ERK1/2 activation enhances survival at later time-points after IR (>24 h) at a time when DNA damage repair is likely to be complete. These data suggest that a mechanism other than DNA repair is responsible for the radiosensitizing effect of selumetinib treatment, consistent with our prior findings (15). In our previous report, we presented data from 3 cell lines with varying levels of sensitization to IR with selumetinib. These data suggest that the presence of a KRAS mutation may increase the efficacy of radiation sensitization observed with selumetinib. To explore the hypothesis that the efficacy of selumetinib as a radiation sensitizer is greater in cells harboring mutant KRAS, we generated a DU145 cell line harboring an activating KRAS mutant. As we expected, the radiosensitizing effects observed with selumetinib were greater in DU145 cells harboring mutant KRAS compared to Ras wild-type cells. However, since we observed a degree of sensitization in the Ras wild-type cells, these data also suggest that the inhibition of the activation of downstream effectors of Ras after IR can sensitize even Ras wild-type cell lines, albeit to a lesser degree.

TGF-α has been well described as a factor that promotes cell proliferation, survival, transformation and protects against radiation-induced damage by activating EGFR downstream intermediates, such as AKT and ERK1/2 (18,21,30). Of note, the transformation of human mammary epithelial cells by the *c-Ha-Ras* gene has been shown to enhance TGF-α expression (31), and the presence of mutant KRAS also promotes TGF-α secretion through TACE activation. Treatment with exogenous TGF-α or conditioned medium collected from cells with oncogenic KRAS has been shown to reverse the radiosensitizing effect of KRAS inhibition (21). Collectively, these former findings suggest that ErbB ligands produced downstream of Ras/MEK/ERK1/2 signaling play an important role in the radiation sensitization obtained with selumetinib in Ras-transformed cells.

The radiation-induced phosphorylation of EGFR and TGF-α secretion coupled with the finding that treatment with a neutralizing TGF-α antibody resulted in radiosensitization. This confirms the importance of TGF-α as a resistance factor to IR, particularly mutant KRAS. With the knowledge that TGF-α is a resistance factor after IR in our cell lines, we investigated the secretion of TGF-α after IR in the setting of treatment with selumetinib. We confirmed that selumetinib reduced TGF-α secretion when delivered alone or in combination with radiation. This suggests that selumetinib may have particular efficacy in tumor cells that rely on basal or inducible TGF-α autocrine signaling. The ability of selumetinib to inhibit TGF-α secretion was confirmed in A549 xenografts, which we have previously shown to be sensitive to selumetinib-induced radiation sensitization (15). Selumetinib treatment diminished the basal levels of TGF-α expression and abrogated an increase following IR. Collectively, these data also suggest that TGF-α expression may be a useful biomarker of drug effects in the setting of radiation sensitization, particularly in KRAS mutant cell lines.

Our data indicate that a possible underlying mechanism for the reduction in TGF-α secretion with selumetinib treatment is the inhibition of the activation of TACE, likely due to a reduction in the association between TACE and phosphorylated ERK1/2. Phosphorylated, active TACE was increased by radiation and decreased by selumetinib treatment in all 3 cell lines. We therefore suggest that the inhibition of pro-TGF-α shedding in irradiated tumor cells treated with MEK inhibition results in the reduction in soluble TGF-α, in turn resulting in the downregulation of the PI3K/AKT pathway. The addition of TGF-α to selumetinib-treated tumor cells following IR restored AKT phosphorylation and partially overcame MEK1/2 inhibition induced by radiation sensitization.

The addition of TGF-α reduced the radiosensitizing effects of MEK1/2 inhibition in all cell lines evaluated. Collectively, these findings suggest that TGF-α is a critical survival factor in KRAS mutant cells and that the radiosensitizing effects of MEK1/2 inhibition are partially related to the inhibition of TGF-α. The rescue of selumetinib and radiation-treated cells after the addition of TGF-α was not complete, suggesting that ERK1/2 activation downstream of TGF-α is partly responsible for its pro-survival effect. Alternatively, it is possible that other unknown pathways and molecules targeted by selumetinib are involved in the radiation sensitizing effect. Additionally, ERK phosphorylation is known to be important in a variety of cellular functions, including cell cycle progression and assembly of the mitotic spindle (32) that are known to be important in the recovery after IR.

In our previous study (15), radiosensitization with selumetinib treatment resulted from mitotic catastrophe rather than apoptosis. We therefore investigated the effects of TGF-α supplementation on 2 known regulators of mitotic catastrophe, Chk2 and survivin. Chk2 phosphorylated on threonine 68 accumulates in BRCA1 nuclear bodies during cell cycle arrest induced by DNA damage (27). Phosphorylated Chk2 facilitated by ATM/ATR in response to radiation-induced DNA damage mediates G2/M cell cycle arrest (33). Of note, the conflict provided by Chk2 inhibition between cell cycle progression and DNA damage could lead to mitotic catastrophe (27). Chk2 has also been shown to correlate with survivin expression and regulate its localization by mediating phosphorylation in response to DNA damage (34–35). Selumetinib treatment diminished most of the radiation-induced Chk2 phosphorylation (Fig. 5C) and enhanced mitotic catastrophe (Fig. 5A) in A549 cells. Survivin is also known to protect against apoptosis and mitotic catastrophe (36,37). When survivin levels are high, cells are protected against drugs that induce apoptosis and mitotic catastrophe. In this study, the level of survivin protein was reduced with selumetinib treatment in irradiated A549 cells and restored by TGF-α. Consistent with these results, the addition of TGF-α to A549 cells treated with selumetinib and radiation, reduced mitotic catastrophe to a level similar to that observed in the untreated irradiated cells.

Taken together, these findings suggest that selumetinib has greater efficacy in KRAS mutant compared to Ras wild-type cells and that this effect may be due to a relatively greater dependence of KRAS mutant cells on TGF-α autocrine signaling following IR. Based on these observations, TGF-α appears to act as a survival factor following radiation, preventing mitotic catastrophe at later time-points via the activation of EGFR. Our results raise the possibility that the radiosensitizing effects of selumetinib may be predicted by determining the dependence of cancer cells on TGF-α after IR.

## Abbreviations:

TGF-α: transforming growth factor-α
ERK1/2: extracellular signal-regulated kinases 1/2
EGFR: epidermal growth factor receptor
MAPK: mitogen-activated protein kinase
MEK1/2: MAPK/extracellular signal-regulated kinase 1/2
TACE: tumor necrosis factor-α converting enzyme
Chk2: checkpoint kinase 2
IR: radiation
TNF-α: tumor necrosis factor-α
NIH: National Institutes of Health
NCI: National Cancer Institute
